# Investigating the Effect of Maltodextrins and Degree of Polymerization on Individual Complex Carbohydrate Taste Sensitivity

**DOI:** 10.1002/fsn3.4751

**Published:** 2025-02-03

**Authors:** Claudia Hartley, Russell S. J. Keast, Wender L. P. Bredie

**Affiliations:** ^1^ CASS Food Research Centre Deakin University Burwood Victoria Australia; ^2^ Department of Food Science University of Copenhagen Frederiksberg C Denmark

**Keywords:** carbohydrate taste, complex carbohydrates, individual taste sensitivity, maltodextrins, sensory, taste perception

## Abstract

Research shows that complex carbohydrates (maltodextrins) can be perceived in the oral cavity independent of sweet taste. However, little is known about individual differences in complex carbohydrate taste sensitivity. Therefore, the relationship between complex carbohydrate structure and individual complex carbohydrate taste sensitivity requires further investigation. This study investigated individual taste sensitivity among adults for maltodextrins with different degrees of polymerization. Participants (*n* = 37) (BMI (kg/m^2^): 24.29 ± 1.06, age (years): 30.32 ± 1.24) taste perception and oral sensitivity for sour (citric acid), sweet (glucose), and complex carbohydrate (mixture of short chain maltodextrins (SCM, average DP 6) and mixture of long chain maltodextrin (LCM, average DP 20)) were assessed using taste assessment measures (detection threshold (DT) and suprathreshold intensity perception (ST)). Taste assessment measures were performed in a randomized, repeated, blinded design. There were significant correlations between LCM DT, SCM DT, Sour DT, and Sweet DT (all *p* < 0.01). There were further significant correlations between LCM ST, SCM ST and Sweet ST (all *p* < 0.01) and between SCM ST, Sweet ST and Sour ST (all *p* < 0.01). There was a significant effect of sex on DT ranking values (*p* = 0.050). For the majority of participants, complex carbohydrate sensitivity status did not change according to chain length. This study strengthens existing research that complex carbohydrates can be perceived in the oral cavity and highlighted that for the majority, maltodextrin chain length does not influence complex carbohydrate taste sensitivity (specifically DT and ST).

## Graphical abstract

Using taste assessment methods, this research investigated individual complex carbohydrate taste sensitivity for two maltodextrins. This research demonstrated that for the majority of participants, maltodextrin degree of polymerization (DP) did not impact complex carbohydrate taste sensitivity. Additionally, these findings support existing research by demonstrating that complex carbohydrates can be detected in the oral cavity.

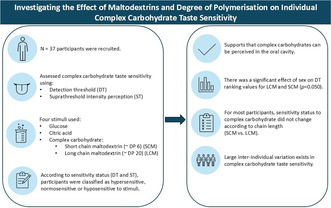

## 1 Introduction

Taste is considered to be one of the conventional five senses (hearing, smell, sight, taste, and touch) (Bachmanov and Beauchamp 2007). According to the current theory, four basic tastes exist: sour, sweet, bitter, and salty (Bachmanov and Beauchamp 2007; McCaughey and Scott 1998) while umami (Hartley, Liem, and Keast 2019; Ikeda 1909), fat (Newman et al. 2016; Stewart et al. 2010; Stewart, Feinle‐Bisset, and Keast 2011; Stewart and Keast 2012) and carbohydrate (Low et al. 2017a) have been proposed to be alimentary tastes (see Hartley, Liem, and Keast 2019 for discussion). Taste is stimulated when taste receptor cells (TRCs), which are situated in the oral cavity, are activated by non‐volatile, saliva‐soluble compounds (Bachmanov and Beauchamp 2007; Hartley, Liem, and Keast 2019). This stimulation enables signal transduction to parts of the brain which then facilitates taste perception (Bachmanov and Beauchamp 2007; Hartley, Liem, and Keast 2019). This taste perception, in combination with input from other senses, will either encourage the consumption or rejection of a food.

While sugars and simplex carbohydrates are identified through the sweet taste receptor T1R2/T1R3 (Nelson et al. 2001; Zhao et al. 2003), it was thought that complex carbohydrates (such as oligosaccharides and polysaccharides) were invisible to the human palate (Feigin, Sclafani, and Sunday 1987; Hettinger, Frank, and Myers 1996). However, recent research demonstrated the presence of independent taste receptors for complex carbohydrates in the oral cavity, independent of sweet taste. Research from Sclafani (1991); (Sclafani 2004) first demonstrated that various rodents (e.g., gerbils, rats, hamsters, and mice) and some nonhuman primates showed an attraction to the taste of complex carbohydrates that originated from maltodextrin. Further research illustrated that humans could also detect complex carbohydrates independently from sugars. Lapis, Penner, and Lim (2016) demonstrated that participants could successfully distinguish glucose oligomer samples from water (Lapis, Penner, and Lim 2016). Furthermore, when Lactisole (a sweet taste inhibitor) was used, the detectability of the glucose oligomers was not compromised (Lapis, Penner, and Lim 2016). Further research used taste assessment methodology (detection threshold (DT) and suprathreshold intensity perception (ST)) to demonstrate that complex carbohydrates (specifically maltodextrin) can be perceived in the oral cavity, over a range of concentrations (Low et al. 2017b, 2019).

Maltodextrin is classified as a complex carbohydrate as it possesses a variable starch‐based structure (Featherstone 2015). Maltodextrins are frequently mixtures of oligomers and polymers consisting of d‐glucose chains which are linked together by glycosidic α‐1,4 and α‐1,6 bonds (Tiefenbacher 2017; Valenzuela and Aguilera 2015). Maltodextrins can differ based on their chemical and physical properties which can subsequently impact the physical and chemical properties (BeMiller 2019). Commercially, maltodextrins are used commonly added to food and beverages as a thickener, stabilizer, and/or sweetener (American Chemical Society 2018). As maltodextrins represent a subtype of complex carbohydrate, this research focuses primarily on maltodextrins and refers to them using the term ‘complex carbohydrates’.

Research has also investigated individual taste sensitivity with complex carbohydrate stimuli. In a randomized, crossover study, participants consumed two isocaloric preload milkshakes (a sweet glucose‐based milkshake or a non‐sweet maltodextrin‐based milkshake) followed by an ad libitum intake of the same drink (Low et al. 2019). Via taste assessment methodology (including DT and ST), participants were classified as either hypersensitive, normosensitive, and hyposensitive to complex carbohydrates (Low et al. 2019). Participants who were more sensitive to maltodextrin consumed a significantly greater quantity of maltodextrin‐based milkshake. This finding was compared to less sensitive participants and this was irrespective of liking (Low et al. 2019). Low et al. (2017a) also demonstrated that having increased sensitivity or experiencing high intensity to complex carbohydrates was significantly associated with a greater energy intake, starch intake, and additionally, a larger waist circumference (Low et al. 2017a). These findings were in comparison to those who experienced a lower intensity or were less sensitive. The effect of complex carbohydrate taste sensitivity on energy intake was further investigated by Costanzo et al. (2021). The authors found that with oligofructose stimuli, according to a 24‐diet record, on average, each decrease in DT concentration step was associated with a 1068 kJ increase in energy intake. This suggests that participants who were more sensitive to complex carbohydrates tended to have a greater daily carbohydrate intake and overall daily energy intake (Costanzo et al. 2021).

Taste properties of maltodextrins can vary according to their branching and chain length. The degree of polymerization (DP) (the number of linked units in a saccharide (Lim and Pullicin 2019)) and dextrose equivalent (DE) (quantity of reducing sugars relative to the total carbohydrate count (Hofman, van Buul, and Brouns 2016)) are a factor that can affect maltodextrin taste properties. For example, longer chain maltodextrins have a lower DE and higher average DP (Birch, Azudin, and Grigor 1991) and viscous mouthfeel (Marchal, Beeftink, and Tramper 1999). In a focus group study performed by Lapis, Penner, and Lim (2016), a higher DP glucose oligomer was described as ‘starchy’, like a root vegetable, pasta, or bread. Conversely, shorter chain maltodextrins with a higher DE and lower average DP are reported to elicit a sweeter taste (BeMiller 2019). Recent research investigated maltodextrins with varying DP and their taste perception (specifically sweetness, starchiness, mouthfeel, and intensity) (Hartley, Keast, et al. 2024). This research determined that the sweetness and intensity of maltodextrins was significantly influenced by maltodextrin DP. Martin et al. (2023) investigated the taste detection of three maltooligosaccharide solutions using a tongue swabbing technique. They demonstrated that maltooligosaccharides (DP 3–20) can be successfully detected by humans (Martin et al. 2023). Similar research conducted by Lapis, Penner, and Lim (2014) investigated individual differences in the taste perception and discrimination of complex carbohydrates. Additional research has investigated individual complex carbohydrate taste sensitivity with oligofructose (Costanzo et al. 2021) and a long chain maltodextrin (DP 24) (Low et al. 2017b). However, at present, no research has investigated individual complex carbohydrate taste sensitivity with multiple maltodextrin samples of varying chain length.

Furthermore, research has demonstrated that a maltodextrin‐based oral rinse can improve exercise performance (Hartley, Carr, et al. 2022). However, existing research in this area has given insufficient attention to the specific maltodextrins (low or high DP) that may improve exercise performance, and this may account for the inconclusive results in the literature. Furthermore, there is a need to obtain more insight into variation in sensory sensitivities among individuals for different maltodextrin types. This further insight into induvial complex carbohydrate taste sensitivity may inform future research involving the use of maltodextrins in nutrition or sports research.

Therefore, based on gaps in the literature, the present study aims to further investigate individual oral sensory sensitivities for complex carbohydrate (maltodextrin). Further, this study aims to investigate how individual complex carbohydrate taste sensitivity differs with maltodextrins of varying chain length using taste assessment methods (detection threshold and suprathreshold intensity perception).

## 2 Methods

### 2.1 Participants

Thirty‐seven participants (BMI (kg/m^2^): 24.29 ± 1.06, age (years): 30.32 ± 1.24) volunteered to complete this study. They were recruited across Copenhagen, Denmark. The inclusion criteria for this study included: (1) participants aged 18–50. Participants were excluded from participation in this study if they: (1) had known food allergies; (2) were smokers; (3) had self‐reported impaired smell or taste function; (4) were pregnant or lactating. To participate in this research, each participant provided their written, informed consent. The protocol was approved by the Deakin University Human Research Ethics Committee and the University of Copenhagen Ethics Committee. Participants were required to fast for minimum 1 h before attending the session.

### 2.2 Experimental Design

Participants attended one session at the Sensory Laboratory at the University of Copenhagen in Copenhagen, Denmark. Data was collected in isolated sensory booths using Compusense Cloud software (part of the Compusense Academic Consortium) (v21, Compusense Inc., Guelph, ON, Canada). Data was generated through accessing research infrastructure at Copenhagen University, including FOODHAY (Food and Health Open Innovation Laboratory, Danish Roadmap for Research Infrastructure). This study followed a blinded, randomized study design. Participants had their taste assessed for four tastants (sweet, sour, and two complex carbohydrates (maltodextrins)). Anthropometric measurements were collected, and participants completed a Food Frequency Questionnaire (FFQ) (McLennan and Podger 1999).

### 2.3 Sensory Stimuli

Two prototypical stimuli (glucose and citric acid) were used to investigate taste function for two basic tastes (sweet and sour) (for further details of stimuli, see Table 1). To investigate oral complex carbohydrate taste sensitivity, two complex carbohydrates (maltodextrins) were used (for further details of stimuli, see Table 1; as described). One sample was a short chain maltodextrin (SCM) (average DP 6), and the second sample was a long chain maltodextrin (LCM) (average DP 20). Before testing, solutions were prepared with filtered drinking water and stored in glass beakers at room temperature (20°C ± 1°C).

**TABLE 1:** Tastant stimuli concentrations used for determination of detection thresholds (DT) for complex carbohydrate (SCM and LCM), sweet (glucose) and sour (citric acid).

Stimulus	Concentration (% w/v)								
	1	2	3	4	5	6	7	8	9
Glucose	0.05	0.09	0.1	0.2	0.4	0.6	1.1	1.8	2.9
Citric acid	0.013	0.016	0.020	0.025	0.031	0.038	0.048	0.06	0.10
SCM	0.1	0.2	0.3	0.6	1.1	1.9	3.6	6.3	11.2
LCM	0.1	0.2	0.3	0.6	1.1	1.9	3.6	6.3	11.2

*Note:* This concentration series was used in previous research (Low et al. 2017b) and based on ISO methods (International Standards Organisation 1991).

Abbreviations: LCM, long chain maltodextrin; SCM, short chain maltodextrin.

### 2.4 Sensory Methods

Each participant's taste function was assessed using a measure of taste perception consistently used in chemosensory research: (1) detection threshold (DT); and (2) suprathreshold intensity perception (ST). These measures were tested for two prototypical tastants (sour (citric acid, Ward McKenzie, VIC, Australia) and sweet (glucose, Melbourne Taste Depot, VIC, Australia)) and two additional tastants (maltodextrins (C*Dry MD 01915 and C*Dry MD 01955, Cargill, France) for oral complex carbohydrate taste measurement)). Two maltodextrins of varying chain lengths were chosen to investigate differences in individual taste sensitivity. As literature is limited in this area, the prototypical tastants of glucose (sweet) and citric acid (sour) were selected to investigate any interaction between sweet taste sensitivity, sour taste sensitivity and complex carbohydrate sensitivity. To ensure accurate taste measures were collected, all measures were repeated for each tastant. All solutions were served in a sample cup at room temperature printed with a three‐digit blinding code for blinding purposes. For DT, participants assessed each taste quality and were given a 5 min break before the next replicate. Participants were then given a further 5 min break before assessing ST.

DT was determined according to previous research (Low et al. 2017b) and procedures based on the International Standard Organization (ISO) Method of Investigating Sensitivity of Taste (International Standards Organisation 1991). Nine concentrations were presented for each taste quality (Table 1). For each tastant, 15 mL samples were presented to each participant, displayed with a three‐digit randomized code and during testing participants wore a nose clip. The nine samples were presented in ascending concentration (i.e., dilution 9 (the weakest concentration) to dilution 1 (the strongest concentration)). For each sample, participants were asked if: (1) the sample tasted like water; (2) something other than water; or (3) a specific taste quality (sour, sweet, bitter, umami, or salty). Participants rinsed their mouth with the filtered water provided between samples as an oral rinsing agent (Low et al. 2017b). The testing of all tastants was repeated to ensure accurate measurements were collected. DT was defined as the concentration upon which participants selected the ‘taste identified, but unknown taste quality’ response (International Standards Organisation 1991).

To determine ST for the two prototypical tastes (sour and sweet) and two complex carbohydrates (maltodextrin) (Table 2), three concentrations (weak, medium, and strong) and a blank (control) sample were used. For every tastant, each participant received a tray with four concentrations and for each sample, the presentation order was randomized. Participants wore a nose clip during testing, and all samples were labeled with a three‐digit randomized code. Participants were instructed to place the 15 mL sample in the mouth and hold it for 5 s before expectorating. Participants rated their perceived intensity of each sample using the Labeled Magnitude Scale (LMS), a psychophysical tool (Green, Shaffer, and Gilmore 1993). This scale is a vertical line scale with descriptors ranging from ‘barely detectable’, ‘weak’, ‘moderate’, ‘strong’, ‘very strong’ and ‘strongest imaginable’. Participants rinsed their mouth with filtered water between samples (Low et al. 2017b). To ensure accurate measurements were collected, the testing of all stimuli for DT and ST was repeated. Prior to receiving samples for DT and ST, participants received a strong, sweet sample as a training sample. This ensured that participants used the correct procedure when tasting the sample, and participants were also able to rate and scale the sample appropriately.

**TABLE 2:** Tastant stimuli concentrations used for determination of suprathreshold intensity (ST) for complex carbohydrate (SCM and LCM), sweet (glucose) and sour (citric acid).

Stimulus	Concentration (% w/v)		
	Weak	Medium	Strong
Glucose	5.3	10.6	21.2
Citric acid	0.02	0.06	0.13
SCM	3.6	6.3	11.2
LCM	3.6	6.3	11.2

*Note:* This concentration series was used in previous research (Low et al. 2017b).

Abbreviations: LCM, long chain maltodextrin; SCM, short chain maltodextrin.

### 2.5 Dietary Assessment

To assess and quantify participant's dietary intake, a Food Frequency Questionnaire (FFQ) was used. This questionnaire was adapted from the 1995 Australian National Nutrition Survey FFQ (McLennan and Podger 1999) and has been used in previous research (Low et al. 2018). The questionnaire was further adapted in order to suit the Danish participants and to avoid confusion. For example, specific Australian food items such as vegemite, twisties and cordial were removed and certain foods were renamed to the appropriate Danish terms (i.e., hot chips to fries; capsicum to bell pepper; and meat pie or sausage roll to savory pastry). In the questionnaire, participants were required to indicate on average, how many times in the previous month they had consumed certain food, beverages, vitamin, and mineral supplements. The questionnaire was comprised of 118 items divided into various categories: bread and cereal foods; meat; dairy foods; vegetables and fruits; sweets, baked goods, and snacks; fish and eggs; dressings; and non‐dairy beverages. On a nine‐point scale, participants selected an answer with response options including ‘never or less than once per month’ to ‘6 or more times per day’. When completing the statistical analysis of the FFQ, the response options for the consumption variables were collapsed in order to conduct the appropriate statistical analyses (Kourouniotis et al. 2016). For example, originally, the white bread category had nine response options and subsequently was recoded down to three response options.

### 2.6 Anthropometry

Participants were instructed to remove any heavy clothing, shoes, and items in pockets before anthropometric measurements were taken. Weight was taken using a digital scale (BWB‐600) (Tanita Corporation, Tokyo, Japan) and height was measured using a portable stadiometer (Leicester Height Measure MKII) (SECA, Hamburg, Germany). BMI was calculated as weight (kg)/height (m^2^) (King 2007).

### 2.7 Statistical Analysis

Statistical analyses were conducted using Stata Statistical software version 16.0 (StataCorp LLC, Texas, USA). Data is presented as mean ± SEM unless stated otherwise. Statistical significance was accepted at *p* < 0.05. Descriptive statistics were used to describe demographic information and DTs and STs for complex carbohydrate, sweet, and sour stimuli. The DTs were calculated as the mean of the repeated measures and the natural log transformation applied. To determine ST, the mean of the three ratings (weak, moderate, and strong) was calculated. DTs and STs for maltodextrin were treated as grouping variables (tertiles) with participants categorized as more sensitive (hypersensitive) (1/3), normal sensitive (normosensitive) (2/3) or less sensitive (hyposensitive) to complex carbohydrates to examine differences between continuous (BMI) and categorical (sex) variables using independent *t*‐tests. Further analysis was completed with a linear mixed model using DT ranking score, sample and sex as fixed variables and the participant as the random variable. DT ranking score was calculated using participant's LCM DT and SCM DT values according to previous research (Costanzo et al. 2018). Paired t‐tests were used to investigate differences between DT values for maltodextrin samples and ST values for maltodextrin samples. To detect differences taste sensitivity status (tertiles) and the frequency of consumption of complex carbohydrate‐based foods, Fisher's Exact test was used. Pearson's product–moment correlation coefficients were used to analyze complex carbohydrate sensitivity (DTs and STs) and BMI. Extreme outliers were identified and removed prior to calculating the Pearson's product–moment correlation coefficient.

## 3 Results

### 3.1 Participants

A total of 37 participants completed this study. This participant pool included 10 males age (years): 31.7 ± 1.8, (range 25–41), BMI (kg/m^2^): 24.8 ± 1.0, (range 20.9–29.7) and 27 females age (years): 29.8 ± 1.6, (range 21–49), BMI (kg/m^2^): 24.1 ± 1.4, (range 15.6–53.2). Overall, four participants were classified as underweight (BMI < 18.5 kg/m^2^) and 11 participants were classified as overweight or obese (BMI > 25 kg/m^2^ (World Health Organization 2000)).

### 3.2 Oral Detection Thresholds

Table 3 shows an overall of mean DT values and ranges for each measured taste quality. When examining the effect of sex on taste sensitivity, data suggests a trend of differences in complex carbohydrate taste sensitivity between males and females. Participant's mean (natural log) DT values show a trend that males may be more sensitive to LCM than females (−1.7 ± 0.4 vs. −0.7 ± 0.3, *p* = 0.0694). The results also suggest a trend towards the same for SCM (−1.8 ± 0.1 vs. −1.0 ± 0.3, *p* = 0.0912). Further analysis using DT ranking methods supports these results and demonstrates that sex has a statistically significant effect on ranking DT values (*p* = 0.05), with a mean DT ranking difference of 1.43 between males and females. Using DT tertile grouping, there were no significant differences in BMI between less and more sensitive participants to complex carbohydrates (LCM: *p* = 0.4293; SCM: *p* = 0.6277). Participants DT values for LCM, sweet, and sour stimuli (Table 3) are consistent with previous research conducted by our research group (Hartley, Carr et al. 2024). Table 4 shows an overview of mean DT values and sensitivity status for each group (tertile) and tastant.

**TABLE 3:** Natural log detection thresholds (DT) (% w/v) for complex carbohydrate (maltodextrin), sweet (glucose) and sour (citric acid) stimuli including mean, SEM and range.

Stimulus	Mean DT ± SEM	Range
SCM	−1.2 ± 0.2	−2.3 to 1.8
LCM	−1.0 ± 0.2	−2.3 to 2.8
Glucose	−1.5 ± 0.4	−3.0 to 0.6
Citric acid	−4.0 ± 0.1	−4.3 to −3.1

Abbreviations: LCM, long chain maltodextrin; SCM, short chain maltodextrin.

**TABLE 4:** Natural log detection thresholds (DT) (% w/v) and sensitivity status for complex carbohydrate (maltodextrin), sweet (glucose) and sour (citric acid) stimuli including mean, SEM and range.

Stimulus	Sensitivity	Number of participants per tertile	Mean DT ± SEM	Range
SCM	Hypersensitive	*n* = 16	−2.1 ± 0.1	−2.3 to −1.9
	Normosensitive	*n* = 9	−1.5 ± 0.0	−1.6 to −1.4
	Hyposensitive	*n* = 12	0.2 ± 0.3	−1.2 to 1.8
LCM	Hypersensitive	*n* = 14	−2.2 ± 0.0	−2.3 to −1.9
	Normosensitive	*n* = 11	−1.3 ± 0.1	−1.6 to −0.9
	Hyposensitive	*n* = 12	0.7 ± 0.4	−0.8 to 2.8
Glucose	Hypersensitive	*n* = 14	−2.8 ± 0.0	−3.0 to −2.7
	Normosensitive	*n* = 11	−1.9 ± 0.1	−2.6 to −1.4
	Hyposensitive	*n* = 12	0.4 ± 0.9	−1.1 to 10.0
Citric acid	Hypersensitive	*n* = 14	−4.3 ± 0.0	−4.3 to −4.2
	Normosensitive	*n* = 11	−4.0 ± 0.0	−4.1 to −3.8
	Hyposensitive	*n* = 12	−3.5 ± 0.1	−3.8 to −3.1

Abbreviations: LCM, long chain maltodextrin; SCM, short chain maltodextrin.

Comparing participants' sensitivity status (DT tertiles) for both complex carbohydrate stimuli (LCM and SCM), the sensitivity status of 51.35% (*n* = 19) participants did not change. Of the 18 participants that did have a change in sensitivity between LCM and SCM stimuli, *n* = 9 had an increase in sensitivity and *n* = 9 had a decrease in sensitivity.

### 3.3 Oral Suprathreshold Intensities

There were no significant differences for mean ST values between males and females (*p* > 0.05). Table 5 shows an overview of mean ST values for each taste quality. Using ST tertile grouping, there were no significant differences in BMI between those who experience low and high intensity to complex carbohydrates (*p* > 0.05). Table 6 displays an overview of mean ST values and sensitivity status for each tastant. There was a statistically significant difference in SCM ST compared to LCM ST (*p* = 0.0001), demonstrating that on average, participants experienced a higher taste intensity with the SCM sample compared to the LCM sample.

**TABLE 5:** Suprathreshold intensity (ST) ratings for complex carbohydrate (maltodextrin), sweet (glucose) and sour (citric acid) stimuli, including mean, SEM and range.

Stimulus	Mean ± SEM	Range
SCM	14 ± 1.7*	1.3–48
LCM	7.3 ± 1.1*	0.0–27
Glucose	44 ± 2.5	19–70
Citric acid	30 ± 1.9	10–57

*Note:* Presented mean of all concentrations (weak, medium and strong).

Abbreviations: LCM, long chain maltodextrin; SCM, short chain maltodextrin.

* Indicates statistically significant difference (*p* < 0.05).

**TABLE 6:** Suprathreshold intensity (ST) ratings and sensitivity status for complex carbohydrate (maltodextrin), sweet (glucose) and sour (citric acid) stimuli, including mean, SEM and range.

Stimulus	Sensitivity	Number of participants per tertile	Mean ST ± SEM	Range
SCM	Hyposensitive	*n* = 13	5.3 ± 0.46	1.3–7.4
	Normosensitive	*n* = 12	11 ± 0.73	7.7–15
	Hypersensitive	*n* = 12	26 ± 2.9	16–48
LCM	Hyposensitive	*n* = 13	2.0 ± 0.28	0.0–3.3
	Normosensitive	*n* = 12	5.3 ± 0.46	3.7–8.3
	Hypersensitive	*n* = 12	15 ± 1.7	8.6–27
Glucose	Hyposensitive	*n* = 13	28 ± 1.7	19–36
	Normosensitive	*n* = 12	45 ± 1.1	38–50
	Hypersensitive	*n* = 12	62 ± 1.6	53–70
Citric acid	Hyposensitive	*n* = 13	18 ± 1.2	10–23
	Normosensitive	*n* = 12	28 ± 0.84	24–34
	Hypersensitive	*n* = 12	44 ± 1.9	35–57

*Note:* Presented mean of all concentrations (weak, medium and strong).

Abbreviations: LCM, long chain maltodextrin; SCM, short chain maltodextrin.

Assessing participants' sensitivity status (ST tertiles) for both complex carbohydrate stimuli (LCM and SCM), the majority (54%, *n* = 20) of participants' sensitivity status did not change. For participants that did have a change in sensitivity between LCM and SCM stimuli, *n* = 9 had an increase in sensitivity and *n* = 8 had a decrease in sensitivity.

### 3.4 Dietary Data

When participants were grouped into tertiles based on complex carbohydrate taste sensitivity (DTs and STs), there were no differences in frequency of consumption of complex carbohydrate‐based foods (*p* > 0.05). There were no significant differences between frequency of consumption of complex carbohydrate‐based foods between males and females (*p* > 0.05).

### 3.5 Correlations

No significant correlations between LCM, SCM DT, or ST for BMI (*p* > 0.05) or body mass (kg) (*p* > 0.05). There was a significant correlation between Sweet ST and BMI (*r* = −0.35, *p* = 0.0339). LCM DT was also significantly correlated with SCM DT, Sour DT, and Sweet DT (displayed in Table 7). There was a significant correlation between SCM DT, Sour DT, and Sweet DT (Table 7). LCM ST was significantly correlated with SCM ST and Sweet ST, whereas there was a significant correlation between SCM ST and Sweet ST and Sour ST (as shown in Table 8). Scatterplots of the correlations are available as Figure S1.

**TABLE 7:** Summary of Pearson's product moment correlation coefficients between natural log detection thresholds for LCM, SCM, Sweet and Sour.

	LCM DT	SCM DT	Sour DT	Sweet DT
LCM DT	—	0.65*	0.60*	0.59*
SCM DT	0.65*	—	0.67*	0.41*

*Note:* Values are Pearson's product moment correlation coefficients (*r* value).

Abbreviations: LCM, long chain maltodextrin; SCM, short chain maltodextrin.

* Indicates statistically significant correlation (*p* < 0.05).

**TABLE 8:** Summary of Pearson's product moment correlation coefficients between LCM ST, SCM ST, Sweet ST and Sour ST.

	LCM ST	SCM ST	Sour ST	Sweet ST
LCM ST	—	0.69*	0.32	0.51*
SCM ST	0.69*	—	0.64*	0.60*

*Note:* Values are Pearson's product moment correlation coefficients (*r* value).

Abbreviations: LCM, long chain maltodextrin; SCM, short chain maltodextrin.

* Indicates statistically significant correlation (*p* < 0.05).

## 4 Discussion

The aim of this study was to investigate complex carbohydrate taste sensitivity among individuals. A secondary aim was to investigate how individual complex carbohydrate taste sensitivity differs with maltodextrins of varying chain length. This research is significant as it adds further insight into sensitivity differences to complex carbohydrates and builds upon previously conducted work by Lapis, Penner, and Lim (2014); Low et al. (2017a); Low et al. (2018, 2019). Furthermore, this research investigates complex carbohydrate taste sensitivity for two maltodextrins of varying chain length. Individual complex carbohydrate taste sensitivity and sensitivity differences among the population are important to consider in future sport and nutrition research involving maltodextrins. As previous work has illustrated that complex carbohydrate taste sensitivity can influence starch intake, anthropometry (Low et al. 2017a) and energy intake (Costanzo et al. 2021; Low et al. 2017a) further research is required. This highlights that future research involving maltodextrins may benefit from investigating participants' sensitivity status either as screening criteria or a post hoc grouping.

At present, this is the first study to suggest a difference in complex carbohydrate taste sensitivity between males and females. For LCM DT, the evidence suggested a trend that male participants were more sensitive than females (*p* = 0.0694). The results also suggested a trend towards the same for SCM DT (*p* = 0.0912). This hypothesis was further supported by results that demonstrated a significant effect of sex on ranking DT values (*p* = 0.050). Previous research has investigated the effect of sex on complex carbohydrate taste sensitivity and found no differences between males and females (Costanzo et al. 2021; Low et al. 2017a, 2017b) or used a female‐only sample group (Low et al. 2018, 2019). Potential differences in complex carbohydrate taste sensitivity based in sex may be due to numerous reasons. Research has demonstrated that hormonal differences can affect taste sensitivity (Alberti‐Fidanza, Fruttini, and Servili 1998; Costanzo 2023). Genetics might also provide an explanation for possible sex‐based differences in carbohydrate taste sensitivity. For example, for umami taste, genetic variation in the TAS1R1/TAS1R3 taste receptor effects umami taste sensitivity (Shigemura et al. 2009). As the specific taste receptor or underlying mechanisms are unknown it is plausible that this genetic variation could also extend to complex carbohydrate taste perception and sensitivity. Furthermore, women were also found to be more insulin sensitive (Geer and Shen 2009) and have a lower resting metabolic rate (Arciero, Goran, and Poehlman 1993) compared to males. This could affect how carbohydrates are utilized and metabolized in the body and potentially effect individual taste sensitivity. Further research should be conducted with a larger, gender balanced study population to examine this further.

Similarly with previous research (Low et al. 2017b, 2018), this study found that substantial inter‐individual variation exists for complex carbohydrate taste sensitivity. For example, for LCM ST, at the strongest concentration of 11.2% w/v, a participant who experienced higher intensity rated the sample at 30.62 on the LMS. Conversely, a participant who experienced lower intensity to the LCM sample rated the sample at 1.29 on the LMS. Additionally, there was large interindividual variation found between DT values across participants. For a less sensitive participant (hyposensitive) with the LCM sample, their DT (natural log) was at 2.6% w/v, while a more sensitive (hypersensitive) participant's DT was at −2.3% w/v, this is a range of roughly 130‐fold. Inter‐individual variation could be due to the previously discussed factor of genetic variation (Shigemura et al. 2009) and additional factors such as fungiform papillae density (FPD) and oral microbiota composition (Cattaneo et al. 2019). Research by Cattaneo et al. (2019) investigated inter‐individual taste variability with basic taste stimuli (sweet, bitter, salty, and sour) and 6‐n‐propylthiuracil (PROP). According to participants' PROP responsiveness, participants were grouped as PROP non‐tasters (less sensitive) or PROP super‐tasters (more sensitive). They found that there was a significance difference in the relative abundance of five bacterial genera with more and less sensitive participants (Cattaneo et al. 2019). Additionally, there was a significant difference in FPD, with less sensitive participants showing reduced FPD compared to more sensitive participants. This highlights that multiple factors may influence taste perception and cause inter‐individual taste variation.

This study also demonstrated that there are potential differences in the taste perception between maltodextrins of varying chain lengths and structure. There was a statistically significant difference in perceived intensity between LCM ST and SCM ST. Participants experienced a higher intensity for the SCM sample (13.7 ± 1.7) in comparison to the LCM sample (7.3 ± 1.1) (*p* = 0.0001). A reason for this difference in intensity could be due to the differences in DE and DP between the samples. In comparison with the LCM sample, the SCM sample is a shorter chain maltodextrin with a higher DE value (DE ~6 vs. DE ~17, respectively). The higher DE value could increase the perception of a ‘sweet‐like’ taste and this could therefore be perceived as more intense than the LCM sample. Due to the taste assessment measures used in this study, it can be argued that the difference in taste perception between samples is due to taste sensitivity to complex carbohydrates and not due to visual or olfaction clues (Low et al. 2018). This is ensured by: (1) participants not consuming any sample and rinsing their mouths with filtered water in between each sample; (2) during testing participants wearing nose clips to minimize orthonasal or retronasal olfaction cues; and (3) samples being prepared on each day of testing (Low et al. 2018).

When examining individual complex carbohydrate taste sensitivity, the majority of participants' individual taste sensitivity status did not change irrespective of sample chain length. For DT and ST values, over half of participants were consistent in their sensitivity status for both the LCM and the SCM samples (51.35% and 54%, respectively). This suggests that regardless of chain length, the taste perception of complex carbohydrates is relatively consistent among individuals. These findings may also indicate that the underlying mechanisms or taste receptor responsible for complex carbohydrate taste perception may not be influenced by the chain length or structure of the stimuli used in this study.

For this research there was no association or relationship between frequency of consumption of complex carbohydrate‐based foods and individual sensitivity status. This is in contrast with previous research where they demonstrated that having an increased sensitivity to complex carbohydrates was associated with a greater energy intake, starch intake, (Low et al. 2017a) and carbohydrate intake (Costanzo et al. 2021). Similarly to this study, Low et al. (2018) found no associations between dietary patterns and complex carbohydrate taste sensitivity and suggest that differences could be attributed to the tools used for dietary assessment. The FFQ that was used assesses individual food products and not overall percentage energy intake and overall starch intake. Low et al. (2018) argue that perhaps there is no difference in frequency of consumption of complex carbohydrate‐based foods in those who are more or less sensitive to complex carbohydrates but rather those who are more sensitive consume a greater quantity (Low et al. 2018). This difference in consumption quantity would not be detected by the FFQ. Future research investigating this area should consider incorporating various dietary measures to collect data on both frequency of consumption and overall percentage energy and starch consumption.

Additionally, this research strengthens the existing literature that complex carbohydrates can be perceived in the oral cavity. Unlike early research that assumed that complex carbohydrates were invisible to the human palate (Feigin, Sclafani, and Sunday 1987; Hettinger, Frank, and Myers 1996), the findings from this study align with previous research that found that humans can sense complex carbohydrates in the oral cavity (Lapis, Penner, and Lim 2014, 2016; Low et al. 2018). Furthermore, the results demonstrated that LCM DT was strongly correlated with SCM DT, Sour DT, and Sweet DT (all *p* < 0.01), and SCM DT was also strongly correlated with Sour DT and Sweet DT (all *p* < 0.01). This in contrast with previous literature where no robust correlations were found between complex carbohydrate DT (maltodextrin and oligofructose) and basic taste stimuli DT (sweet, sour, salty or bitter) (Low et al. 2017b). As it has been demonstrated that complex carbohydrates are perceived independently from sweet taste (Lapis, Penner, and Lim 2016), the correlation in DT between complex carbohydrates (LCM and SCM), sweet and sour stimuli suggest that this may be due to the participants themselves. As the majority of participants that completed this research had participated in sensory testing (specifically descriptive analysis and sensory panels) previously, they likely had been selected for their higher sensory acuity (Kilcast 2004) which could have contributed to the correlation.

Furthermore, as LCM ST and SCM ST were strongly correlated (*p* < 0.01), these findings highlight that maltodextrins between DP 6–20 may share similar transduction pathways for taste perception. This is similar to previous research that found strong correlations between maltodextrin and oligofructose samples and hypothesized that similarities in transduction pathways exist (Low et al. 2017b). Interestingly, LCM ST was strongly correlated with SCM ST and Sweet ST (all *p* < 0.01), and SCM ST was correlated with Sweet ST and Sour ST (all *p* < 0.01). This is consistent to findings in previous research (Low et al. 2017b). Low et al. (2017b) hypothesize that a novel taste receptor could nonetheless be involved in the detection of complex carbohydrates but simply at the detection range. At ranges where perception occurs (at ST concentration levels), the authors speculate that complex carbohydrate perception may be partially facilitated by the T1R‐independent sweet sensing pathways and the theorized complex carbohydrate detection receptor (Low et al. 2017b). Similarly, Lapis, Penner, and Lim (2016) hypothesize that for glucose oligomers specifically, it is plausible that a T1R‐independent sweet sensing pathway enables perception of glucose oligomers via their hydrolysis to glucose. However, this theory would not account for the independent detection of polysaccharides in the oral cavity (Lapis, Penner, and Lim 2016). Further research is required to investigate the underlying mechanisms and potential receptors for complex carbohydrate taste perception.

### 4.1 Considerations

For this study, DT was calculated according to International Standards Organisation (1991). DT was defined as the concentration upon which participants selected the ‘taste identified, but unknown taste quality’ response (International Standards Organisation 1991). This method has also been used by previous research (Hartley, Carr, et al. 2024; Hartley, Costanzo, et al. 2022; Hartley et al. 2023; Low et al. 2017b, 2019, 2017). The authors acknowledge that due to this method, the DT may be slightly overestimated. The alternative method of calculating the geometric mean proposed by Lawless and Heymann (2010) may result in a slightly lower DT value.

Furthermore, in the participant group of this study, four participants were classified as underweight (BMI 15.6–18.4 kg/m^2^) and one participant was classified as extremely or morbidly obese (BMI > 40 kg/m^2^) (Purnell 2018). Due to this, the authors discussed that these participants could potentially be regarded as outliers based on their BMI. However, on closer examination, all participants were spread across different sensitivity groups (tertiles) for both LCM DT and ST and SCM DT and ST. For example, according to DT, one participant was classified as normosensitive to LCM stimuli yet hyposensitive to SCM stimuli, and another participant was classified as hyposensitive to LCM stimuli and hypersensitive to SCM stimuli. This therefore disputes the idea of these participants as outliers and supports the inclusion of the data points in the analysis. Furthermore, to include a diverse range of individuals in this study, participants were not excluded from the study based on their body mass or BMI. Excluding participants based on weight status may introduce selection bias and could compromise the study's scientific integrity. Participants have often been excluded from human‐based research based on BMI or weight status and this has resulted in underrepresentation in research (Pagarkar, Harrop, and Erlanger 2023).

### 4.2 Limitations

Despite using thorough methodology, some limitations exist with this research. During sample testing, this study did not control for salivary α‐amylase. Salivary α‐amylase hydrolyses α‐1,4 glycosidic bonds in carbohydrates and starches causing the production of maltose, maltotriose, and larger oligosaccharides (Mandel et al. 2010). However, previous research has suggested that the hydrolysis byproducts are likely not at a level that is perceptible in the oral cavity (Hartley, Keast, et al. 2024; Lapis, Penner, and Lim 2014).

## 5 Conclusion

In conclusion, this study highlighted that individual complex carbohydrate taste sensitivity is multifaceted. For the majority of participants, individual complex carbohydrate taste sensitivity (specifically DT and ST) was not affected by maltodextrin chain length. Furthermore, this study supports existing research by demonstrating that complex carbohydrates can be detected in the oral cavity. This study also demonstrated that large inter‐individual variation exists in complex carbohydrate taste sensitivity. Therefore, this highlights that individual sensitivity is critical to investigate for future research, especially sports and nutrition research involving complex carbohydrates.

## Author Contributions

**Claudia Hartley:** conceptualization (equal), data curation (lead), formal analysis (lead), investigation (lead), methodology (equal), project administration (lead), resources (lead), visualization (lead), writing – original draft (lead), writing – review and editing (equal). **Russell S. J. Keast:** conceptualization (equal), methodology (equal), supervision (equal), writing – review and editing (equal). **Wender L. P. Bredie:** conceptualization (equal), investigation (supporting), methodology (equal), supervision (equal), writing – review and editing (equal).

## Ethics Statement

This study received ethical approval from the Deakin University Research Ethics Committee and assigned the ethics number: 2022–281. This study also received ethical approval from the University of Copenhagen and was assigned the ethics number: 514–0356/22–5000. The application was approved on 5/12/2022.

## Consent

All subjects were provided with a Plain Language Statement and gave their written, informed consent to participate in this research.

## Conflicts of Interest

The authors declare no conflicts of interest.

## Supporting information


Figure S1.

## Data Availability

The datasets generated during and/or analyzed during the current study are available from the corresponding author on reasonable request.
