# Supplementary material for: Investigating the Effect of Maltodextrins and Degree of Polymerization on Individual Complex Carbohydrate Taste Sensitivity
Source: Food Sci Nutr. 2025 Feb 3;13(2):e4751. doi: 10.1002/fsn3.4751 (PMC11790608; doi:10.1002/fsn3.4751)
Supplement: Supplementary file 1 — Figure S1. [file FSN3-13-e4751-s001.docx]

**
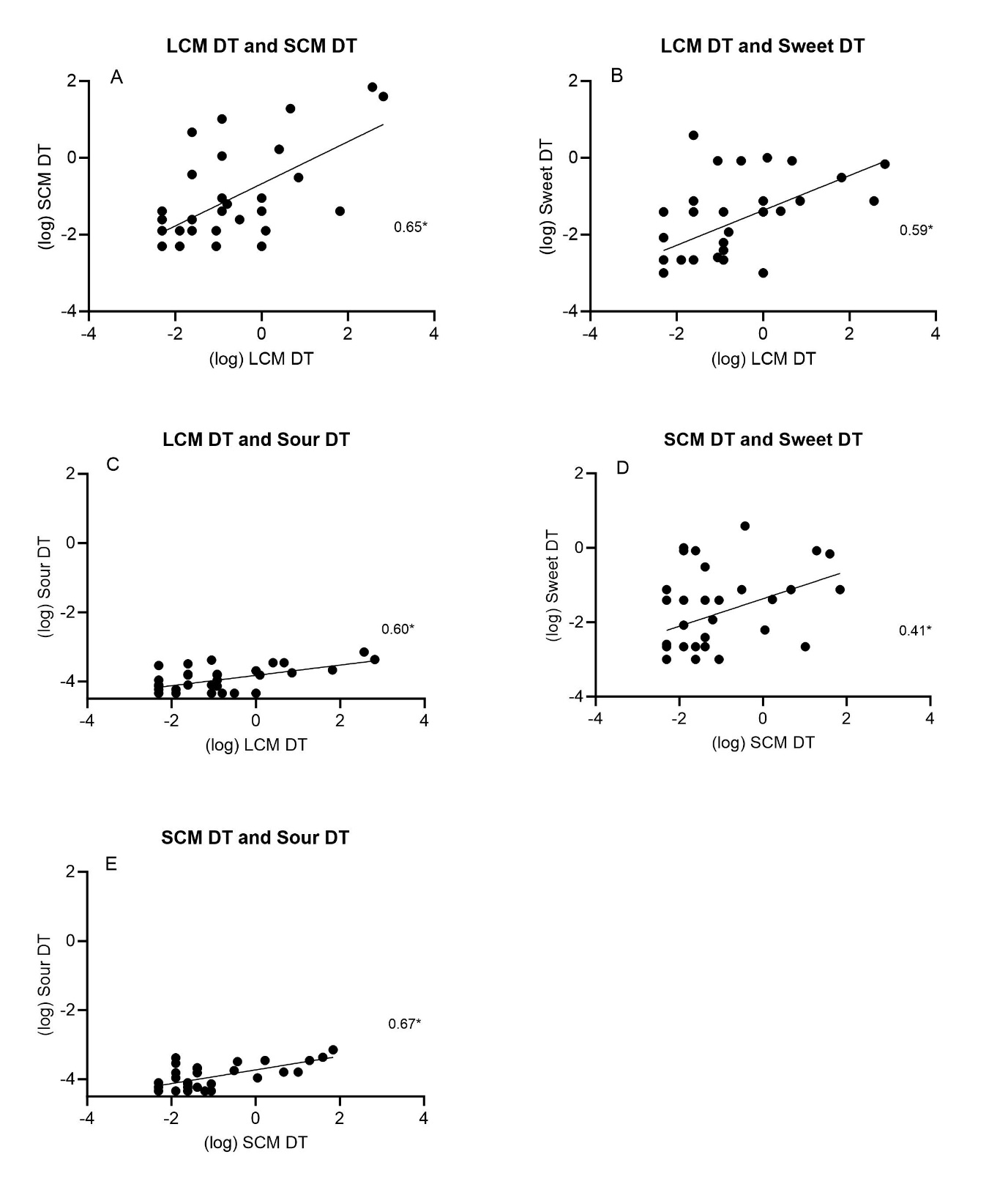
Supplementary Figure 1.** Scatterplots and Pearson correlations (r value) between natural log detection thresholds (DT) of complex carbohydrates (long chain maltodextrin (LCM) and short chain maltodextrin (SCM)), sweet and sour stimuli. (A) Correlations between LCM DT and SCM DT. (B) Correlations between LCM DT and Sweet DT. (C) Correlations between LCM DT and Sour DT. (D) Correlations between SCM DT and Sweet DT. (E) Correlations between SCM DT and Sour DT. * Indicates statistically significant correlation (p < 0.05).

**
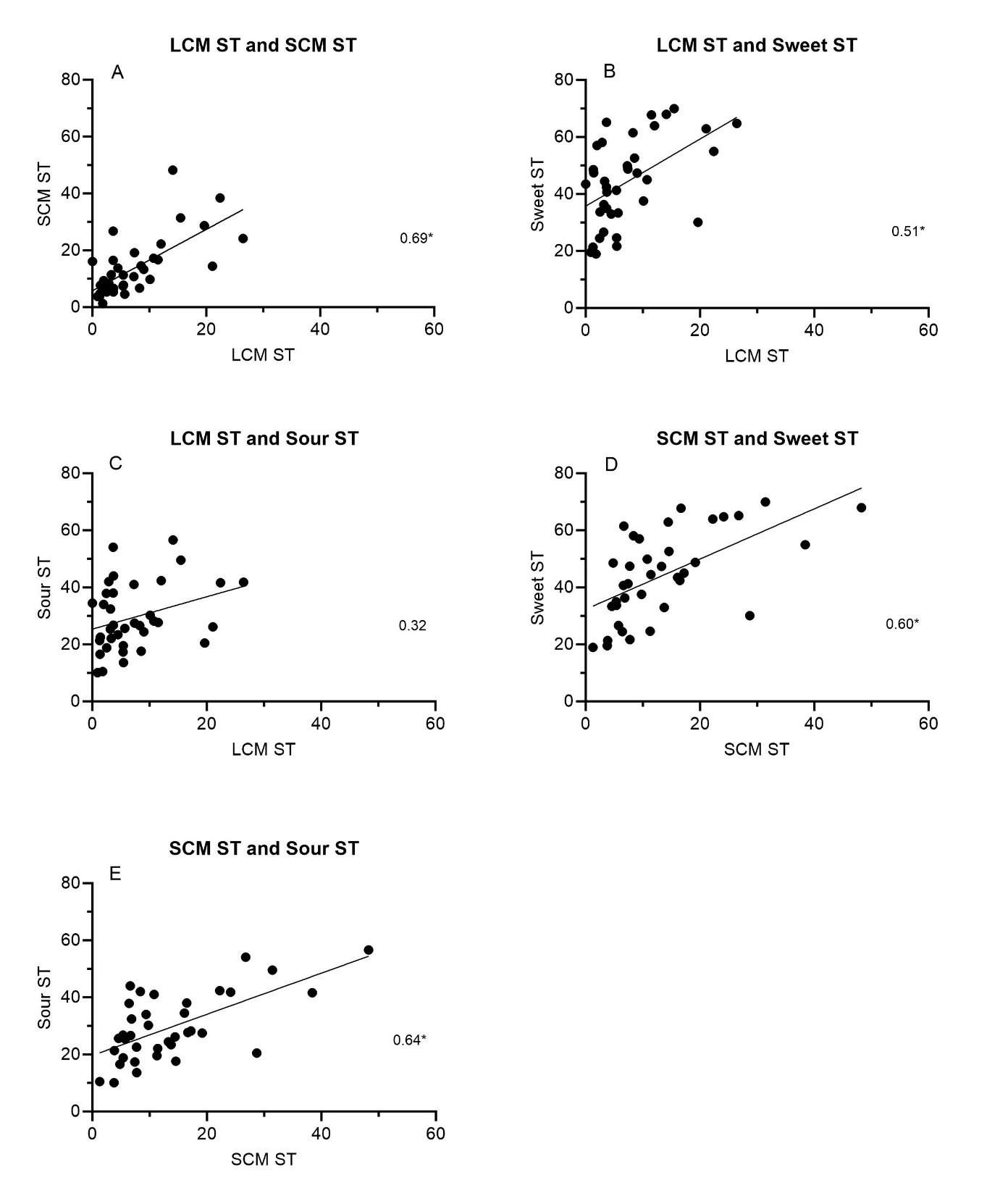
Supplementary Figure 2.** Scatterplots and Pearson correlations (r value) between suprathreshold intensity perception (ST) of complex carbohydrates (long chain maltodextrin (LCM) and short chain maltodextrin (SCM)), sweet and sour stimuli. (A) Correlations between LCM ST and SCM ST. (B) Correlations between LCM ST and Sweet ST. (C) Correlations between LCM ST and Sour ST. (D) Correlations between SCM ST and Sweet ST. (E) Correlations between SCM ST and Sour ST. * Indicates statistically significant correlation (p < 0.05).
